# Supplementary material for: Coevolutionary dynamics in the grass-livestock social-ecological system of China’s alpine pastoral areas: A case study of the Qilian Mountains region in China
Source: PLoS One. 2025 Jan 30;20(1):e0317769. doi: 10.1371/journal.pone.0317769 (PMC11781735; doi:10.1371/journal.pone.0317769)
Supplement: S1 Appendix — (DOCX) [file pone.0317769.s001.docx]

# S1 Appendix. Model Construction of the Dynamic Simulation for the Grass-Livestock System in China’s Alpine Pastoral Areas.

**S1.1 Forage-livestock module**

The forage-livestock module simulates the relationship between forage yield per hectare of grassland and livestock weight (**Fig 1**). Their interaction is mainly connected through the stocking rate (Stocking rate), intake (Intake), and degradation effect index (Degradation effect). Relevant theories indicate that different grazing intensities have varying impacts on the net primary productivity of grasslands[^[[1]](#endnote-0)^][^[[2]](#endnote-1)^]. In lightly grazed areas, the high residual amount of forage affects the growth of forage during the regreening period. Moderately grazed areas have good forage regeneration, coupled with the "compensatory growth" of livestock at the beginning of grazing, which is beneficial for livestock weight gain. In overgrazed areas, forage growth exhibits a “lag effect”. During the early and middle stages of grazing, forage growth and regeneration are strong, and the forage growth and regeneration can meet the livestock's intake needs. However, in the later stages of grazing, forage growth and regeneration decline, and the forage yield cannot meet the livestock's intake needs, leading to a decrease in livestock weight.


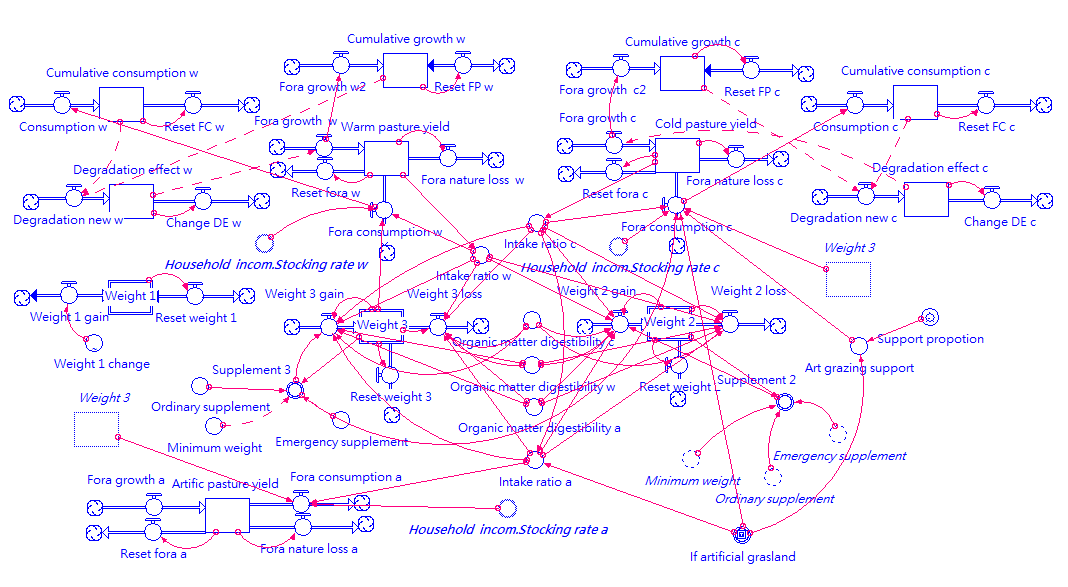


**Fig 1. Simulation model of grassland herbage yield-livestock weight module.**

In the model, pasture yield per hectare (Pasture yield) is directly proportional to forage growth (Forage growth) and inversely proportional to forage consumption (Forage consumption) and forage nature loss (Forage nature loss). Forage growth and forage nature loss are calculated based on the maximum net primary productivity during the peak growing season and the monthly dynamic changes in local forage yield [^[[3]](#endnote-2)^] (forage yield data sourced from monitoring data of the Qilian County Grassland Monitoring Bureau). Forage consumption is calculated from intake and stocking rate, the calculation formula is as follows:

*Forage consumption = Weight×intake ×Stocking rate*30.*

According to grazing experiment research results [^[[4]](#endnote-3)^], when the net primary productivity of grassland per hectare falls below 200 kg/hm², livestock intake decreases with the net primary productivity of the grassland, the calculation formula is as follows: *Intake=0.00018*pasture_yield.*

When the net primary productivity of the grassland exceeds 200 kg/hm², the intake (Intake refers to the feed intake per kilogram of metabolic body weight, with the unit of g·W^0.75^·kg^-1^·d^-1^.) and organic matter digestibility (Organic matter digestibility) per kilogram of metabolic body weight for each season are set according to relevant experimental literature [^[[5]](#endnote-4)^] [^[[6]](#endnote-5)^].

The weight (Weight) of adult livestock is directly proportional to organic matter digestibility and intake. According to relevant experimental research [^[[7]](#endnote-6)^], if the intake of organic matter is less than the minimum required to maintain weight, the livestock will lose weight; otherwise, they will gain weight, the calculation formula: *Weight gain/loss=±0.82((Weight×Intake)×Organic_matter_digestibilitye-0.0268×Weight0.75)/(0.2036×Weight0.75).*

According to supplementary feeding survey data, the supplementary feeding period is from December to May of the following year. In the model, various types of forage supplements are uniformly converted into hay supplements (Supplement) based on price, with each sheep requiring 5.51 kg of hay per month (Ordinary supplement). If the weight of adult livestock falls below the minimum weight (Minimum weight), which is set at 20 kg, they are considered dead. To prevent death from excessively low weight, pastoralists will purchase a large amount of emergency forage to ensure livestock survival.

After forage is consumed, its regeneration performance mainly depends on the net effect between promotion and inhibition. Here, we introduce the degradation effect index (Degradation effect), which ranges from 0 to 1. A lower value indicates more severe degradation, with 0 representing complete degradation and 1 representing no degradation. The formula for calculating the index is determined based on forage monitoring experimental data from adjacent areas [^[[8]](#endnote-7)^]. If the ratio of cumulative consumption (Cumulative consumption) to cumulative growth (Cumulative growth） of forage per hectare during the growing season is ≤0.23, it indicates no degradation; otherwise, degradation occurs, and the larger the ratio, the more severe the degradation, the calculation formula: *Degradation effect=-1.6398×(Cumulative_consumption/Cumulative_growth)+1.389229.*

**S1.2 Livestock production management module**

**(1) Pastoral Household Expected Livestock Numbers Module**

The pastoral household expected livestock numbers module calculates the total cost and profit of livestock production for each year and determines productive investment based on the profit (**Fig 2**). By constructing a Cobb–Douglas production function, it calculates the maximum number of stocking livestock under the level of labour, capital and pasture area in that year, which can also be considered as the number of livestock desired by the herder.

*
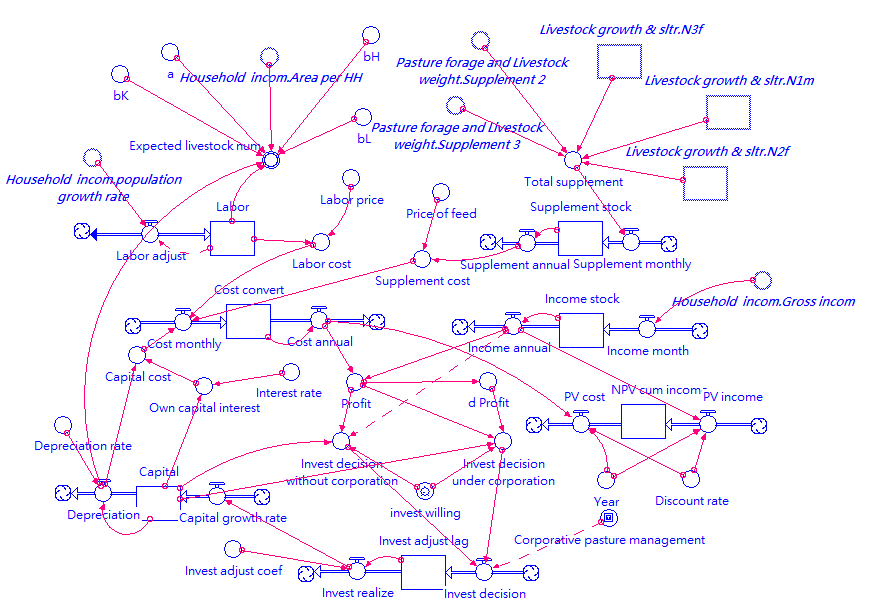
***Fig 2. Simulation model of Livestock production management module.**

Labor (Labor) includes the household labor of pastoralists. Through field research and observation, it was found that local pastoral households conduct small-scale family production by means of household labor and without employing hired workers. The labor price (Labor Price) is set at the local average wage of 3,500 RMB per month.

From December to May of the following year, local pastoralists mainly use mixed concentrates and a small amount of alfalfa, oat grass, and silage corn as feed. In the model, the purchase quantities of these feeds are converted into hay equivalents based on price, mainly supplementing breeding ewes and off-take lambs (Total supplement). The price of feed (Price of feed) is calculated at 1.8 RMB/kg based on the local hay price, the calculation formula: *Total supplement=Supplement_2×N2f+Supplement_3×(N3f+ N1m).*

Capital stock (Capital) refers to productive fixed assets, including pens, machinery, and houses. According to research, the average capital stock per household is 131,125 RMB. Capital stock (Capital) is directly proportional to productive investment (Invest realize) and inversely proportional to depreciation (Depreciation). Fixed capital cost (Capital cost) includes depreciation (Depreciation) and own capital interest (Own capital interest). The model sets the depreciation period at 10 years, applying the double declining balance method to calculate the fixed depreciation rate. The monthly depreciation rate (Depreciation rate) is 1.67%, and the own capital interest rate (Interest rate) is based on the bank deposit interest rate, calculated at 3.5% per annum., the calculation formula*: Capital cost= Depreciation+ Own capital interest.*

Total annual cost (Cost annual) includes labor cost (Labor Cost), supplement cost (Supplement Cost), and fixed capital cost (Capital cost), the calculation formula is as follows:

*Cost annual= Labor Cost+ Supplement Cost+ Capital cost.*

Operating profit (Profit) is the difference between the annual operating income (Income annual) and the total annual cost (Cost annual). Pastoralists will allocate a certain percentage of the profit as productive investment (Invest realize), based on survey data, the investment ratio is set at 0.272.

As independent producers, pastoralists aim to maximize output given the current input levels (Expected livestock numbers), which means the expected livestock numbers. If the current number of livestock (Adjust number of livestock) is less than the expected livestock numbers under the current input levels, pastoralists will increase herd size by reducing off-take. Conversely, if the current livestock numbers exceed the maximum output, pastoralists will off-take excess livestock.

Based on survey data, a Cobb-Douglas production function is constructed. The output variable is expected livestock numbers (Expected livestock numbers), and the input variables include depreciation of fixed assets (Depreciation), labor (Labor), and pasture area per household (Area per household). The model is expressed as *Q=A·L^α^·K^β^·H^δ^*. Taking logarithms on both sides and using the least squares estimation. The results are shown in **Table 1**.

**Table 1. Regression results.**

| Explanatory variable | Regression coefficient | Standard deviation | T-statistic | P-value |
| --- | --- | --- | --- | --- |
| Labor | 0.43 | 0.04 | 10.77 | 0.000 |
| Depreciation of fixed assets | 0.17 | 0.054 | 3.15 | 0.002 |
| Pasture area | 0.40 | 0.03 | 13.91 | 0.000 |
| Constant term | 1.69 | 0.39 | 4.32 | 0.000 |
| R-squared | 88.8% | | | |

**(2) Livestock Expansion and Off-take Decision Module**

The livestock expansion and off-take decision module calculate the annual livestock numbers. Livestock herd growth follows breeding patterns and is influenced by the pastoral household's expected output, determined by their off-take decisions (**Fig 3**). Livestock herds are categorized by age and sex: 1-year-old ewes (N1f), 2-year-old ewes (N2f), 3-7-year-old ewes (N3f), 1-year-old rams (N1m), 2-year-old rams (N2m), and 3-7-year-old rams (N3m). According to field research and observation, the usable lifespan for ewes and rams is 7 years, with the herd structure as follows: N1f: N2f: N3f: N1m: N2m: N3m = 0.165: 0.084: 0.42: 0.165: 0.023: 0.08.

*
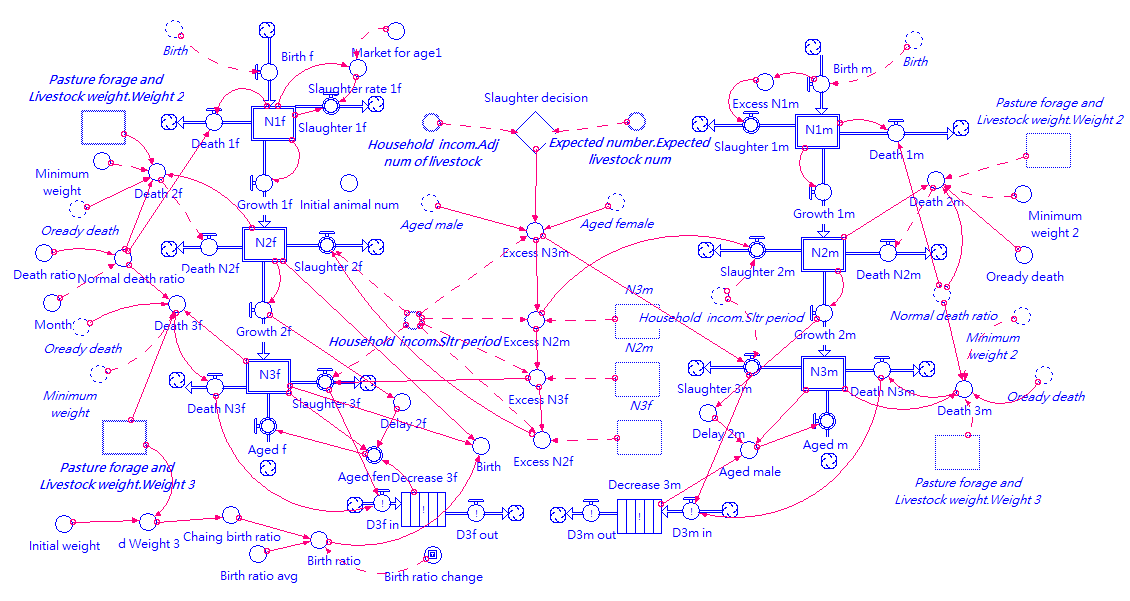
*

**Fig 3. Simulation Module of Livestock Expansion and Off-take Decision.**

The number of breeding ewes (N2f) and (N3f) determines the number of lambs born. Livestock numbers increase due to births (Birth) and decrease due to deaths and off-take (Slaughter). If some 1-year-old ewes (N1f) continue to be retained (Growth 1f) after reaching 12 months, they become 2-year-old ewes (N2f) in the following year. This progression applies similarly to other age groups of ewes and rams. In the model, the birth ratio(Birth ratio)is the ratio of successfully born lambs to the number of breeding ewes, with its dynamic changes depending on two factors: the average birth rate calculated based on local survey data, which shows an average birth rate (Birth ratio average) of 76%, and the influence of grassland conditions on ewe weight, subsequently affecting the birth ratio. Each 1% decrease in ewe weight results in a 0.404% decrease in the birth ratio [^[[9]](#endnote-8)^], the calculation formula is as follow:

*Birth ratio =(Weight_3-Initial_weight)/Initial_weight ×0.404+ Birth_ratio_avg*.

The number of lambs born (Birth) is equal to the number of breeding ewes multiplied by the birth ratio (Birth ratio), the calculation formula: *Birth = (N3f+ N2f)×Birth_ratio*. Local livestock deaths mainly occur from December to March of the following year. According to research, the winter mortality rate is 5.2%, thus setting the monthly winter mortality rate as one-fourth of the total winter mortality rate.

Local pastoralists annually off-take most 1-year-old rams. According to survey data, each year pastoral households retain 46% of the rams for personal consumption and replacing older rams, with the remaining 54% being off-taken. Pastoral household off-take decisions are categorized into two scenarios:

1). If the actual livestock numbers exceed the expected livestock numbers, pastoralists first take 7-year-old cull sheep. If, after off-taking the cull sheep, the actual number still exceeds the expected number, they off-take rams older than 3 years. If, after off-taking rams older than 3 years, the actual livestock numbers still exceed the expected number, they off-take 2-year-old rams, followed by the remaining 1-year-old rams. Finally, they off-take ewes in descending order of age until the actual livestock numbers are less than or equal to the expected livestock numbers.

2. If the actual livestock numbers are less than the expected livestock numbers, they only need to off-take old cull sheep and some 1-year-old rams. The number of 7-year-old cull ewes (Age female) is calculated from the number of 2-year-old ewes 5 years earlier (Delay 2f) and the 3-7-year-old ewes that died or were off-taken over the 5 years (Decrease 3f) , the calculation formula: *Age female =Delay_2f-Decrease_3f/5.*

If the simulation period is less than 5 years, it is represented by dividing the total number of 3-7-year-old ewes (N3f) by 5. The calculation for culling rams follows the same principle.

**S1.3 Pastoralist Income Module**

The pastoral household income module calculates the income earned by pastoralists from managing their pastures and livestock (**Fig 4**). In the alpine pastoral region, the sources of pastoral household income (Gross income) include revenue from the sale of beef and mutton (Meat production value), wool (Wool production value), and yak milk (Milk production value). Among these, the sale of beef and mutton is the primary source of income. The meat production value is calculated as the product of the number of animals off-taken and their live weight at off-take. A conversion coefficient (F to M) is introduced based on the price ratio to eliminate price differences between rams and ewes, with the coefficient set at 0.76 according to the price ratio, the calculation formula is as follows:

*Slaughter weight total = Slaughter_1m× Weight_1+ Slaughter_2m× Weight_2*

*+Slaughter_3m+ Aged_m)× Weight_3+( Slaughter_2f× Weight_2+( Slaughter_3f*

*+ Aged_f)× Weight_3)×FtoM.*

(1) The price of meat (Meat price) for Tibetan sheep is 35 RMB/kg.

(2) The price of wool (Wool price) is 3.5 RMB/kg, with an average wool yield of 2 kg per adult Tibetan sheep.

(3) The price of milk (Milk price) for yak is approximately 10 RMB/kg. The milking period (Milking period) is around 4 months, during which each yak produces about 144.94 kg of milk for sale. However, local pastoralists rarely sell milk, likely due to the underdeveloped yak milk industry in the region. Therefore, the initial value for the milk market (Milk market) is set to 0 in the model.

*
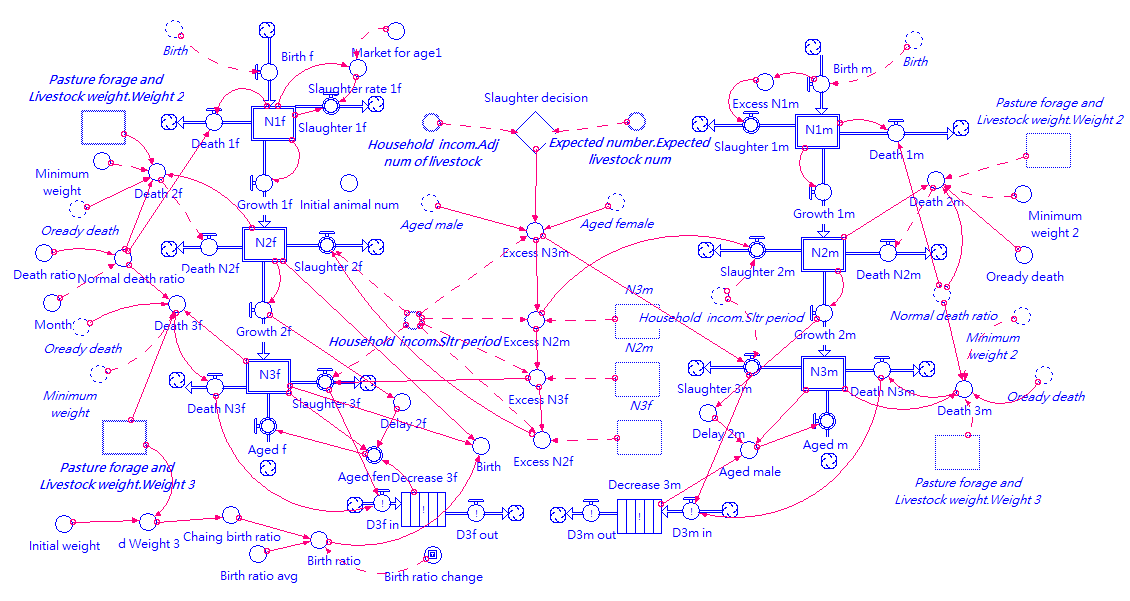
***Fig 4. Simulation Model of Pastoralist Income.**

Household gross profit (Profit) is calculated as the difference between gross income and operating costs, which include feed costs (Supplement Cost), labor wages (Labor Cost), and fixed capital depreciation (Capital Cost).

Pastoral household needs to set grazing areas and corresponding rotational grazing periods. The model sets the area per household (Area per HH) according to the total grazing area (Grazing area) averaged among local households (Household). The phenomenon of family members splitting into separate households further reduces the grassland area per household, so the population growth rate (Population growth rate) is introduced. According to the 2010 Qinghai Province Census data, the natural population growth rate of Qilian County is 0.591%, which is used to represent the growth rate of pastoral households, the calculation formula is as follow:

*Household =Initial_household_number×(1+population_growth_rate)^Year.*

Grazing density is calculated in Tibetan sheep units, determining the stocking rate for cold-season natural grasslands (Stocking rate c), warm-season natural grasslands (Stocking rate w), and artificial grasslands (Stocking rate a). The calculated stocking rate is applied to the forage-livestock module. The number of livestock per household (Adjust number of livestock) is adjusted based on the weight ratios of different ages and sexes, the calculation formula is as follow: *N1f×Rel_weight_1_2f+ N2f×1+ N3f×Rel_weight_3_2f.*

The total adjusted livestock numbers (Total livestock adj in pastoral) are calculated by multiplying the number of households (Household) by the number of livestock per typical household (Adjusted number of livestock). According to data from the Qilian County Agriculture and Animal Husbandry Bureau, there are 602 pastoral households.

**S1.4 Model Data Collection**

| Model Variables | Initial value | Unit |
| --- | --- | --- |
| Weight of Tibetan sheep 25-72 months | 42 | Kg/Pc |
| Number of livestock in a typical pastoral household | 616 | One |
| Number of pastoral households in the study area | 602 | Household |
| Average area per households | 2762/184.13 | Mu/Ha |
| Fixed capital of a typical pastoral household | 131125 | Yuan (RMB) |
| Labor force of a typical pastoral household | 2.5 | One |
| Labor Price | 3500 | Yuan (RMB) |
| Emergency supplement | 30 | Kg |
| Ordinary supplement | 5.51 | Kg |
| Price of feed | 1.8 | RMB/kg |
| Investment ratio | 27.2 | % |
| Invest willing | 40 | % |
| Depreciation rate | 1.67 | % |
| Interest rate | 3.5 | % |
| Birth ratio average | 76 | % |
| Death ratio | 5.2 | % |
| Meat price | 34 | RMB/kg |
| Wool price | 3.5 | RMB/kg |
| Milk price | 10 | RMB/kg |
| F to M conversion coefficient | 76 | % |
| Each yak milk production | 144.94 | kg |
| Proportion cattle | 49 | % |
| Proportion sheep | 51 | % |
| Weight of Tibetan sheep 0-12 months | 6.94 | kg |
| Artifical grassland proportion | 10 | % |
| Clod grassland proportion | 50 | % |
| Warm proportion | 16 | % |
| Summer grassland_proportion | 23 | % |
| Total grassland area | 110867.52 | Mu |
| Rel weight 1to2m | 52.2 | % |
| Rel weight 1-2f | 63.4 | % |
| Rel weight 3to2m | 135.4 | % |
| Rel weight 3-2f | 135.3 | % |
| Organic matter digestibility in artifical grassland | 0.5193 | One |
| Organic matter digestibility in cold grassland | 0.5587 | One |
| Organic matter digestibility in warm grassland | 0.6482 | One |
| Initial artific pasture yield | 300 | Mu/kg |
| Initial cold pasture yield | 100 | Mu/kg |
| Initial warm pasture yield | 100 | Mu/kg |
| Initial degradation effect of cold pasture | 0.8 | One |
| Initial degradation effect of warm pasture | 0.5 | One |

1. **References**

   [] Sun Yi. Interactions of soil, herbage and livestock in the alpine meadow - Tibetan sheep grazing system on the Qinghai-Tibetan Plateau. Lanzhou University, 2015. [↑](#endnote-ref-0)
2. []. Shiping W, Yanfen W, Yonghong L, Zuozhong C. The influence of different stocking rates on herbage regrowth and aboveground net primary production. Acta Agrestia Sinica. 1998;(04): 275-281. [↑](#endnote-ref-1)
3. []. Fupin Z, Huwei W, Yiwen Z, Zhizhi Z, Xiaojuan L. Study on aboveground biomass of natural grassland and balance between forage and Livestock in Qilian County. Journal of Natural Resources. 2017;32(07): 1183-1192. [↑](#endnote-ref-2)
4. []. Shindo K (2015). Grazing Experiment To Determine Animal Intake And Affect On Biomass And Vegetation Of Forest Steppe zone in Mongolia: the results and future target of the research. Japan: Japan International Rearch Center for Agriculture Sciences. [↑](#endnote-ref-3)
5. []. Ting J, Tiecheng W, Jianping W, Shengguo Z, Zhaomin L, Liang Jianyong,et al. A comparative study on digestibility and feed intake of Tibetan sheep of different types . ActaPrataculturae Sinica. 2019;28(05): 100-108. [↑](#endnote-ref-4)
6. []. Yuxin L, Jianzhou W, Long L, Honghui W, Lockzhu L, Qiangba Yangzong,et al. Research of grazing sheep Feed intake and digestibility in northern Tibet cold pastoral in different seasons. Acta Ecologiae Animalis Domastici.2009;30(05):41-45. [↑](#endnote-ref-5)
7. []. TAkahashi T, Jones R, Kemp D R. Steady-state modelling for better understanding of current livestock production systems and for exploring optimal short-term strategies. Australia: Australian Centre for International Agricultural Research. 2021 [↑](#endnote-ref-6)
8. []. Hongtao L, Wei Z, Quanfei D, Yushou M, Jianjun S, Shihai Y. Effects of different grazing intensities on aboveground net primary productivity and regrowth biomass in alpine-cold grassland around Qinghai Lake. Chinese Qinghai Journal of Animal and Veterinary Sciences. 2011; 41(04): 13-15. [↑](#endnote-ref-7)
9. []. Coop I E. Liveweight-productivity relationships in sheep. New Zealand Journal of Agricultural Research. 1962;5(3): 249-264. [↑](#endnote-ref-8)
