# Supplementary material for: Coevolutionary dynamics in the grass-livestock social-ecological system of China’s alpine pastoral areas: A case study of the Qilian Mountains region in China
Source: PLoS One. 2025 Jan 30;20(1):e0317769. doi: 10.1371/journal.pone.0317769 (PMC11781735; doi:10.1371/journal.pone.0317769)
Supplement: Attachment — Submitted filename: Response to Reviewers.docx [file pone.0317769.s003.docx]

# Response to Reviewers for manuscript PONE-D-24-30315

Dear Editors and Reviewer:

On behalf of all contributing authors, I would like to express our sincere appreciation for your letter and the reviewer’s constructive comments concerning our paper entitled “Coevolutionary Dynamics in the Grass-Livestock Social-Ecological System of China’s Alpine Pastoral Areas: A Case Study of the Qilian Mountains Region in China” (PONE-D-24-30315). These comments are valuable and helpful for improving our paper. In response to the editor and reviewers' comments, we have made extensive modifications to our manuscript to enhance the credibility of our results.

This letter is divided into three main parts. The first part addresses the journal's requirements, the second part responds to Reviewer 1’s comments, and the third part responds to Reviewer 2’s comments.

In the revised version of the manuscript, all changes to our manuscript are highlighted in yellow-shaded text. Point-by-point responses to the editor and reviewers are listed below this letter.

lf there are any other modifications we could make, we would like very much to modify them and we really appreciate your help.

Thank you for your consideration.

Sincerely,

Ligan Cai, Junhao Zhao, Jian Chen

# 1. Responses to journal requirements:

*(1) When submitting your revision, we need you to address these additional requirements.*

*Please ensure that your manuscript meets PLOS ONE's style requirements, including those for file naming. The PLOS ONE style templates can be found at*

*https://journals.plos.org/plosone/s/file?id=wjVg/PLOSOne_formatting_sample_main_body.pdf and*

*<https://journals.plos.org/plosone/s/file?id=ba62/PLOSOne_formatting_sample_title_authors_affiliations.pdf.>*

**Response:**

Thank you very much for your reminder. We have made corrections according to the required format.

*(2). Please match your authorship list in your manuscript file and in the system.*

**Response:**

Thank you very much for your reminder. We have reviewed the author list as required.

*(3). You indicated that ethical approval was not necessary for your study. We understand that the framework for ethical oversight requirements for studies of this type may differ depending on the setting and we would appreciate some further clarification regarding your research. Could you please provide further details on why your study is exempt from the need for approval and confirmation from your institutional review board or research ethics committee (e.g., in the form of a letter or email correspondence) that ethics review was not necessary for this study? Please include a copy of the correspondence as an “Other" file.*

**Response:**

Thank you very much for your reminder and understanding. We have briefly described this in the clarification letter and provided confirmation from the Research Ethics Committee that no ethical review was required for this study. Since the first author’s affiliated institution at the time of writing this paper did not have an Institutional Review Board, a well-known psychiatric hospital in China was entrusted to conduct the review, and they issued a confirmation that ethical review was not required.

# 2. Response to reviewer 1’s comments:

**Reviewer #1commented：**(1) The research is potentially interesting but lacks reference to a theoretical framework and research data is somewhat poorly supported. In the title and introduction reference to socio-ecological system is reported, but actually the work is based on a bio-economic model. The most important issue is that the authors do not include any kind of stakeholder participatory round. You may refer to this paper to understand what a socioecological approach is (one of many examples) Elsawah, S., Pierce, S.A., Hamilton, S.H., Van Delden, H., Haase, D., Elmahdi, A. and Jakeman, A.J., 2017. An overview of the system dynamics process for integrated modelling of socio-ecological systems: Lessons on good modelling practice from five case studies. Environmental Modelling & Software, 93, pp.127-145.

**Response:**

We sincerely appreciate your insightful comments, which have significantly contributed to improving our manuscript. Below, we address each of your suggestions:

a.Regarding the lack of theoretical framework:

We acknowledge the insufficiency in referencing a theoretical framework. With guidance from your suggested literature, particularly Elsawah et al. (2017), we have further explored integrated assessment models (IAMs) within social-ecological systems research. We have systematically reviewed relevant literature on the grass-livestock integrated assessment model and expanded Section 2 of the paper, now titled ‘Grass-Livestock Social-Ecological System’. This section clearly outlines the concepts of grass-livestock systems, their role in decision-support studies, and the application of system dynamics (SD) modeling within such systems.

b.Regarding the research data's insufficient support:

We revisited and restructured our data sources to strengthen the empirical support for our arguments. In *Section 3.2.1: Data Source and Sample Selection*, we provide a detailed description and categorization of data sources. Through a survey, we collected data on grassland area types, livestock scales and species, herd age structures, and livestock performance indicators (e.g., body weight, lambing rates, milk production, and mortality). Additional data on production costs, household labor, and fixed assets were obtained from 72 questionnaires, of which 30 were used for constructing representative pastoral household profiles based on averages. Livestock performance data, including intake rates and digestibility, were sourced from relevant literature.

c. Regarding alignment between the title, introduction, and the model:
We understand your concern regarding the discrepancy between the title, introduction, and the bio-economic model used in our study. In response, we have clarified that integrating grassland ecological indicators and livestock biological performance better reflects the ecological-economic interactions within the grass-livestock system. The newly expanded *Section 2* explicitly frames the grass-livestock system as a complex system incorporating nutritional needs, grazing behavior, vegetation ecology, and economic outcomes. We have further emphasized in *Section 2.2* the role of system dynamics (SD) modeling in integrating ecological, biological, and economic parameters for decision-making. This ensures a stronger coherence between the title, introduction, and core content of the paper.

d. Regarding the absence of stakeholder participation:

We fully acknowledge the importance of incorporating stakeholder perspectives, as you highlighted. In fact, during the fieldwork phase, we conducted in-depth observations of key stakeholders, including herding families and government officials, in a manner that did not interfere with or influence their public behavior. These observations include their daily decision-making processes, their intentions to cooperate, and their ways of addressing grassland degradation, and are often representative of prevailing local practices and traditional customs. It could also be noted that this study has built a typical pasture, which blurred the identity information and personality characteristics of the pastoral households. Production and operation data adopt the average number of construction methods. The performance of livestock performance and grassland data present the common characteristics of the research area, the source is a channel for public available acquisition. The common decision-making characteristics formed by the herd households in the management of grazing can be summarized with the help of observation and can be verified by conducting interviews with these objective, common and observable behaviors with the pastoral households. We integrate this data into model development and refinement to better reflect real-world macro-phenomena and stakeholder priorities. Our study is also based on these anonymized data in order to construct a System Dynamics (SD) model of grassland ecology and pastoralism in alpine pastoral areas, and that SD model focuses more on specifying general scenarios and typical changes in Social-Ecological systems rather than focusing on the micro-level behavior of individual pastoralists.

**Reviewer #1commented:**（2）The results corroborate general statement about the need to support cooperative management and sustainable use of grassland resources but I'm wondering if we need a model to support this general statement. The authors should add more details on the data that they used, how was the questionnaire built, haw many years of grass data collection have been performed. The 'mechanics' of the model seems fine but results really depend on the quality of data.For instance, we do not have a clue about the variability of your sample. Thus, it is complicate to understand how an averaged typical farm can be considered a valid approach in the model.

**Response:**

Thank you for your thoughtful comments and for highlighting key areas for improvement. We greatly appreciate your constructive feedback.

a. Regarding the necessity of the model to support general statements:

While it may appear that our results confirm general assertions about sustainable grassland resource use, the model serves a critical role in providing a structured analytical framework. Rather than restating general conclusions, our model delves into the intricate relationships and dynamic interactions among various elements within the grassland resource system. By simulating the long-term effects of different management strategies, such as changes in ecological conditions, livestock productivity, and socio-economic factors, the model offers precise, detailed, and forward-looking insights. This depth and scope far exceed what general statements can achieve and significantly support policymakers in formulating targeted policies and optimizing practical solutions.

b. Regarding additional data details:

We fully agree with your suggestion and will add more specific details about our data collection in *Section 3.2: Data Source* and *S1 Appendix.* This will include details on the questionnaire design, structure, and the years of data collection.

c. Regarding sample variability:

We recognize your concerns about the representativeness of an average typical farm. In our study, the construction of a "typical pastoral household" reflects common characteristics in livestock performance, grassland biomass, and household decision-making behaviors, minimizing variability in these factors. The primary variability stems from differences in grassland area. To address this, we conducted a variability analysis focusing on pasture size. The grassland area in our study region ranges from [33.33 ha～285.33 ha] (mean 184.13, standard deviation 33.73, coefficient of variation 0.183). We selected households with pasture sizes close to the mean, specifically within the range of [150.4 ha～217.87ha] , resulting in 72 representative households. This approach reduces variability in production and operational data, enabling us to construct a more representative typical household. Further details are now clarified in *Section 3.2.2: Analysis of Sample Variability and Representativeness.*

**Reviewer #1commented：**（3）the references to existing data, theoretical framework, etc. is not sufficient and is a clear weakness of the manuscript.

**Response:**

Thank you for pointing out this weakness. To address this, we have conducted a comprehensive review of relevant literature and have systematically integrated these references into the manuscript.

**Reviewer #1commented：**（4）some more specific comments as following:

“L50 intro an indication if ha of pastoral area would be useful as you mention significant influence at globe level”

**Response:**

Thank you for your suggestion. We have added the following information: ‘China's 400 million hectares of pastoral land rank as the world's second-largest, covering approximately 40% of the country. These lands are located in the drier, higher regions of north and northwest China and are primarily inhabited by various ethnic minorities.’

**Reviewer #1commented：**L57 reasons for degradation of these ecosystems?

**Response:**

Thank you for your valuable suggestion. We have added the following: ‘The transformation of China’s alpine pastoral areas is currently facing many challenges, especially due to the irrational number of grazing animals and the imperfect system of grassland resource management, the alpine pastoral areas are faced with the problem of the continuous deterioration of grassland ecology.’

**Reviewer #1commented：**“L62 state is a bit too generic. Is there a specific agency or ministry? A specific law framework?”

**Response:**

Thank you for your suggestion. We have added the following: ‘The State Council, the Ministry of Agriculture and Rural Affairs, the State Forestry and Grassland Administration, and other departments have gradually established a grassland management system. This system is based on the grassland contract responsibility system, grazing bans, artificial grassland construction, and other major projects, with supporting laws, regulations, and policy measures implemented.’

**Reviewer #1commented：**“L81 the first reference appears here after a number of statements. The information reported should be supported by appropriate references.”

**Response:**

Thank you for your comment. Relevant references have now been cited.

**Reviewer #1commented：**“L99 'Violating pastoralists' please, clarify the meaning”

**Response:**

Thank you for your insightful comment. The original sentence, "Violating pastoralists" means pastoralists who violate internal management rules, has been removed in the revised version as we believe it was redundant and did not add value to the context.

**Reviewer #1commented：**“L100 'this new mechanism of joint management can solve the "tragedy of the commons": but at L75 you have already said that ' This policy, by defining property rights, effectively solved the "tragedy of the commons'. In addition, the 'tragedy of the commons' deserves a proper definition and you have not indicated references.”

**Response:**

Thank you for your thoughtful suggestion. The reference to the "tragedy of the commons" has been removed in the revised version, as this phenomenon has not been observed in alpine pastoral areas. The grassland contract system was primarily introduced to enhance household production and facilitate management. We believe that mentioning the "tragedy of the commons" is not essential in this context and does not impact the readers' understanding of the research background.

**Reviewer #1commented：**“L120-124 here you define artificial grassland but I find the description not informative. What is it? Forage crops? renaturalization with commercial seeds? Which species are employed? Statements like 'Artificial grasslands mainly include artificial grasslands for controlling degraded grasslands and semi-artificial grasslands for improving and reseeding natural grasslands' does not help to understand this practice.”

**Response:**

Thank you for your valuable suggestion. We have added the following: ‘Artificial grasslands include areas for growing forage crops for livestock and areas where natural grasslands are improved by reseeding with native and selected commercial species. Their creation aims to boost forage production, control soil erosion in degraded grasslands, and improve the ecological balance of the pasture ecosystem.’

**Reviewer #1commented：**“L134 Please, add a brief explanation (with references) of what a system dynamic approach is.”

**Response**

Thank you for your suggestion. A brief explanation has been added in Section 2.2: Application of System Dynamics (SD) Modeling in Decision Support for Grass-Livestock Systems.We have added the following:‘System Dynamics (SD) modeling is one of the common methods in Integrated Assessment Modeling (IAM). It is a simulation and prediction method that can integrate various key elements and form internal feedback. The SD modeling method can provide a favorable tool for the complex grass-livestock (or pasture) systems and the decision-making needs of stakeholders. It is capable of integrating multidisciplinary knowledge to explain, predict and evaluate the socioeconomic and ecological environment of the grass-livestock system.’

**Reviewer #1commented：**“L130-158 Here you mix gaps in the state of the art and the objectives, but it is not very clear what are the problems and how you intend to tackle them. I would appreciate to have a clear paragraph on what are the problems, gaps of research etc. and then a paragraph to explain the objectives of the study.”

**Response**

Thank you for your insightful feedback. We have recognized the lack of clarity in presenting the research gaps and objectives, which made it difficult to identify the problem and our approach to addressing it. To address this, we have thoroughly reviewed and rewritten the relevant sections.

Firstly, in Section 1: Introduction, we have clarified the research gaps and issues by summarizing the evolution of grassland management policies and highlighting the current research status and challenges, along with stating the research objectives. We have added the following:‘Building on a series of issues identified within the current grassland management policy framework, this study takes China’s alpine pastoral regions as its primary focus. Specifically, it examines the effectiveness of herders’ cooperative organizations, whether timely adjustments to grazing prohibition policies are warranted, and the efficacy of artificial grassland interventions. By addressing these questions, we aim to develop an optimized pathway that harmonizes grassland ecological governance with the economic advancement of alpine pastoral animal husbandry, ultimately providing guidance for policy refinements and sustainable production management in these areas.’

Secondly, in Section 2: Grass-Livestock Social-Ecological System, we emphasized the necessity of applying an integrated assessment of the grass-livestock social-ecological system. We have added the following:‘ Given the complexity of the system, it is necessary to evaluate the grass-livestock system by integrating the knowledge of multiple disciplines such as ecology, economics and biology.However, most of the evaluation methods are non-dynamic, fail to reflect the internal relationships, and are unable to mirror the future changing trends. Faced with the dual contradictions of the degradation of grassland resources and the social and economic development in pastoral areas, stakeholders such as the government and herder households need to evaluate the management strategies and expected results.The design and implementation of effective environmental policies need to be informed by a holistic understanding of the system processes (biophysical, social and economic), their complex interactions, and how they respond to various changes.This poses certain challenges and difficulties for the establishment of predictive and evaluative models.’

Thirdly, in Section 2: Grass-Livestock Social-Ecological System, this section systematically reviews relevant literature on integrated assessments, identifies research gaps, and outlines the research methodology and objectives at the end of this section. We have added the following: ‘For the above reasons, this study chooses to use the system dynamics method to establish a socio-ecological simulation model for the grass-livestock system in alpine pastoral areas, construct the feedback interactions between the biophysical and socio-economic systems , enrich the research on system dynamics simulation in the pasture field, introduce variables related to local grassland management policies, aiming to predict and evaluate the implementation effects of policies and herders’ decisions, so as to support the optimized adjustment of local policy measures.’

**Reviewer #1commented：**“L190 which % of total households do the 72 households cover?”

**Response**

Thank you for your comment. In fact, the 72 households represent 12% of the total 602 pastoral households. We focused on households with pasture sizes close to the mean, specifically within the range of [2256 ha–3268 ha], which included 409 households. From this group, 72 households were randomly selected for the questionnaire survey. This has been clarified in Section 3.2.1: Data Source and Sample Selection (1) Field research.

**Reviewer #1commented：**“L191 '30 typical households were selected from these 72 households' based on what?”

**Response**

Thank you for your suggestion. The production and operational data were derived from the selected 30 households. Since production efficiency largely depends on producers with normal labor capacity, we excluded households with impaired productivity due to illness or disability. Additionally, for historical validation of the model, we required data from ten years ago, thus excluding households that recently began livestock production. Ultimately, we selected 30 representative households with over 10 years of grazing experience and constructed the typical production data for the study area based on averages. This has been clarified in Section 3.2.1: Data Source and Sample Selection (2) Construction of Production and Management Data.

**Reviewer #1commented：**“L202 which experimental data? Reference?”

**Response**

Thank you for your comment. The relevant references have been added in Section 3.2.1: Data Source and Sample Selection (3) Livestock Production Performance Data.

**Reviewer #1commented：**“Table 1 point 4 'pen and circle' What is it?”

**Response**

Thank you for your suggestion. To improve the clarity and conciseness of the manuscript, we have appropriately reduced the number of tables, and Table 1 from the original version has been removed.

**Reviewer #1commented：**“L215 'ONIKI S and PARSONS' why uppercase?”

**Response**

Thank you for pointing this out. The error has been corrected in Section 4.2 Model Development, We have added the following:‘Among these, Parsons (2011) and Oniki (2018) provided rigorous approaches at the level of variable logic architecture, playing an extremely critical role in the construction of the variable logic system in this model.’

**Reviewer #1commented：**“L218 Yak is a small ruminant?”

**Response**

Thank you for your comment, and we apologize for the error in this statement. The section has been removed, and a concise introduction to the model is now presented in Section 2: Grass-Livestock Social-Ecological System.We have added the following:‘ this study chooses to use the system dynamics method to establish a socio-ecological simulation model for the grass-livestock system in alpine pastoral areas, construct the feedback interactions between the biophysical and socio-economic systems , enrich the research on system dynamics simulation in the pasture field, introduce variables related to local grassland management policies, aiming to predict and evaluate the implementation effects of policies and herders’ decisions, so as to support the optimized adjustment of local policy measures.’

**Reviewer #1commented：**“Fig 3 I suggest putting this in appendix and add here a more simplified figure to explain the model. Fig 4, 5 and 6 idem”

**Response**

Thank you for your valuable suggestion. We have moved Figures 3, 4, 5, and 6 to S1 Appendix and added a simplified figure in the main text to present the key logical relationships between variables (Fig 3).

**Reviewer #1commented：**“L267 'Relevant theories' which theories? Defined by whom?”

**Response**

Thank you for your suggestion. A more detailed description of the model has been moved to S1 Appendix, where we have also added relevant references.

**Reviewer #1commented：**“L325 'research' Which research?” and “L373 'research' Which research?”

**Response**

Thank you for pointing this out. The original statement led to a misunderstanding and has been corrected. In **S1 Appendix**, we have rephrased it as: ‘Through field research and observation, it was found that local pastoral households conduct small-scale family production by means of household labor and without employing hired workers.'

**Reviewer #1commented：**“in general, I'd reduce consistently Chapter 3. The full description can be moved in an appendix.”

**Response**

Thank you for highlighting this issue. The original statement caused ambiguity and has been corrected. In S1 Appendix, we now clearly state: ‘According to field research and observation, the usable lifespan for ewes and rams is 7 years, with the herd structure as follows: N1f: N2f: N3f: N1m: N2m: N3m = 0.165: 0.084: 0.42: 0.165: 0.023: 0.08.’ This is based on data collected from household questionnaires and further verified through interviews.

Thank you for your suggestion. Chapter 3 has been streamlined, and the full description has been moved to S1 Appendix for clarity and conciseness.

# 3. Response to reviewer 2’s comments:

**Reviewer #2commented:** (1) Many studies have explored the effects of government management on the Qinghai-Tibet Plateau (e.g., doi:10.1016/j.scitotenv.2024.176404; doi:10.3390/plants12183182; DOI: 10.3389/fpls.2022.991287; DOI: 10.1002/ldr.3835), but this paper did not mention the contributions of these studies. It is recommended to supplement the literature review to better position the innovation of this study.

**Response**

Thank you very much for pointing out the shortcomings in our literature review. The relevant literature you recommended is highly valuable for improving the research context. We have thoroughly reviewed these studies and incorporated their findings into the ‘2. Grass-Livestock Social-Ecological System’ section as needed. This addition allows us to more accurately position the innovation of our study within the field and highlight its distinctions and unique contributions compared to existing research.

**Reviewer #2commented:** (2) There are too many figures in the main text. It is suggested to merge or delete some figures to focus on presenting the most important results.

**Response**

Thank you for your valuable suggestions. We have incorporated your feedback by moving **Figu 3, 4, 5,** and **6** to S1 Appendix and adding a simplified version of **Fig 3** in the main text. Additionally, **Table 1** and **Table 5** have been removed, and the detailed model description, along with some figures and tables, has been relocated to S1 Appendix to reduce the number of figures and tables in the main text.

**Reviewer #2commented: (3)** The number of references is relatively small. It is recommended to add more relevant high-quality references to enhance the scientific rigor of the study.

**Response**

Thank you for your insightful suggestion. We fully agree that incorporating more high-quality references can significantly enhance the scientific rigor and robustness of the study. We have conducted a comprehensive review of relevant literature, and in addition to the previously cited works, we have studied additional key references in this research field. These references have now been integrated into the Introduction, Research Methods, and sections providing supplementary explanations of relevant theories and techniques.

**Reviewer #2commented:** (4) Minor Comments

1. The figures are rather rough, especially **Figure 1**. It is recommended to further refine them.

**Response**

Thank you for your suggestion. The map has been improved, and we have also added photographs of the research sites to provide a clearer and more comprehensive representation.

**Reviewer #2commented:** 2. The description "See S1" is not specific enough; it is recommended to clarify it.

**Response**

Thank you for your clarification. In compliance with the journal’s formatting requirements, S1 Appendix refers to **Appendix 1**, as the journal allows authors to use nearly any description as the item name for a supporting information file as long as it contains an “S” and number.

This paper includes two appendices:

- S1 Appendix: Model Construction of the Dynamic Simulation for the Grass-Livestock System in China's Alpine Pastoral Areas.
- S2 Appendix: Data on the dynamic changes in average profit of pastoralists, livestock weight, numbers, and grassland forage yield from 2022 to 2042.

The supporting information captions are listed at the end of the manuscript, and the full content can be accessed in the supporting information files.

**Reviewer #2commented:** 3.Figure 3 only has a title; it is suggested to add a brief explanation in the main text.

**Response**

Thank you for your suggestion. In the 4.2 Model Development section, we have added the following explanation: ‘The grassland pasture yield-livestock weight module simulates the relationship between pasture yield per hectare of grassland and livestock weight; their mutual influence is primarily linked through grazing rate, intake, and degradation impact index’. Additionally, we have provided a brief description of Fig 3: Simulation model of grassland herbage yield-livestock weight module.

**Reviewer #2commented:** 4. The text in Figures 7, 8, 9, and 10 is too small, and it is recommended to enlarge the font.

**Response**

Thank you for your suggestion. We have increased the font size in Fig 7, 8, 9, and 10 for improved readability.

**Reviewer #2commented:** 5. The spelling of "Decisio" is incorrect and should be changed to "Decision."

**Response**

Thank you for your suggestion. The changes have been made accordingly.

**Reviewer #2commented:** 6. The use of "livestock number" and "livestock numbers" should be consistent.

**Response**

Thank you for your suggestion. The revisions have been completed.

**Reviewer #2commented:** 7. The sentence "the number of livestock remain high" should be changed to "the number of livestock remains high" to ensure subject-verb agreement.

**Response**

Thank you for your feedback. The corrections have been made.

We would like to express our sincere gratitude for the valuable time and effort you have dedicated to the review process. Your insights and suggestions are greatly appreciated.

Yours sincerely,

The authors of this paper
